# Cell-laden biomimetic microneedles reconstruct skin rete ridge and stem cell niche

**DOI:** 10.1186/s12951-025-03430-x

**Published:** 2025-06-04

**Authors:** Xiaohong Zhao, Zongze Wu, Yicheng Guo, Lei Pu, Zixuan Pei, Yuanyuan Liu, Biao Hou, Songlin Xie, Gaoxing Luo, Rixing Zhan

**Affiliations:** 1 Institute of Burn Research, State Key Laboratory of Trauma and Chemical Poisoning, Southwest Hospital, Third Military Medical University (Army Medical University), Chongqing, 400038 China; 2 Department of Hand and Foot Microsurgery, The affiliated Nanhua Hospital, Hengyang Medical College, University of South China, Hengyang, 421002 China

**Keywords:** Wound repair, Rete ridges, Bionic microneedles, Stem cell niche

## Abstract

The traditional skin graft wound repair strategy for burns is unable to reconstruct the normal anatomy of the skin, resulting in quality problems such as scarring, which have always been the bottleneck of burn medicine. The skin rete ridge (RRs) is an important basis for maintaining skin homeostasis, but the complete reconstruction of the RRs in wound repair is still difficult with traditional split-thickness skin grafting and wound cell therapy. In this study, based on our previous experience of wound epidermal stem cell (EpiSCs) therapy, we further designed and optimized the bionic RRs microneedles loaded with human EpiSCs (C-Ms). Transplanting C-Ms into full-thickness wounds in nude mice promotes the formation of RRs similar to natural human epidermis and provides stem cell niches. Pathway enrichment analysis showed that C-Ms transplantation into wounds reshapes the extracellular matrix (ECM) and reduces wound healing fibrosis through FAK, ECM receptor interaction signaling pathways. Moreover, C-Ms improve wound healing by accelerating early vascularization, and they also regulate the proliferation of EpiSCs to further promote the formation of RRs structure. These results suggest that C-Ms can compensate for the lack of skin anatomical structure, which may be suitable for use in clinical patients with large-scale burns.

**Graphical abstract:**

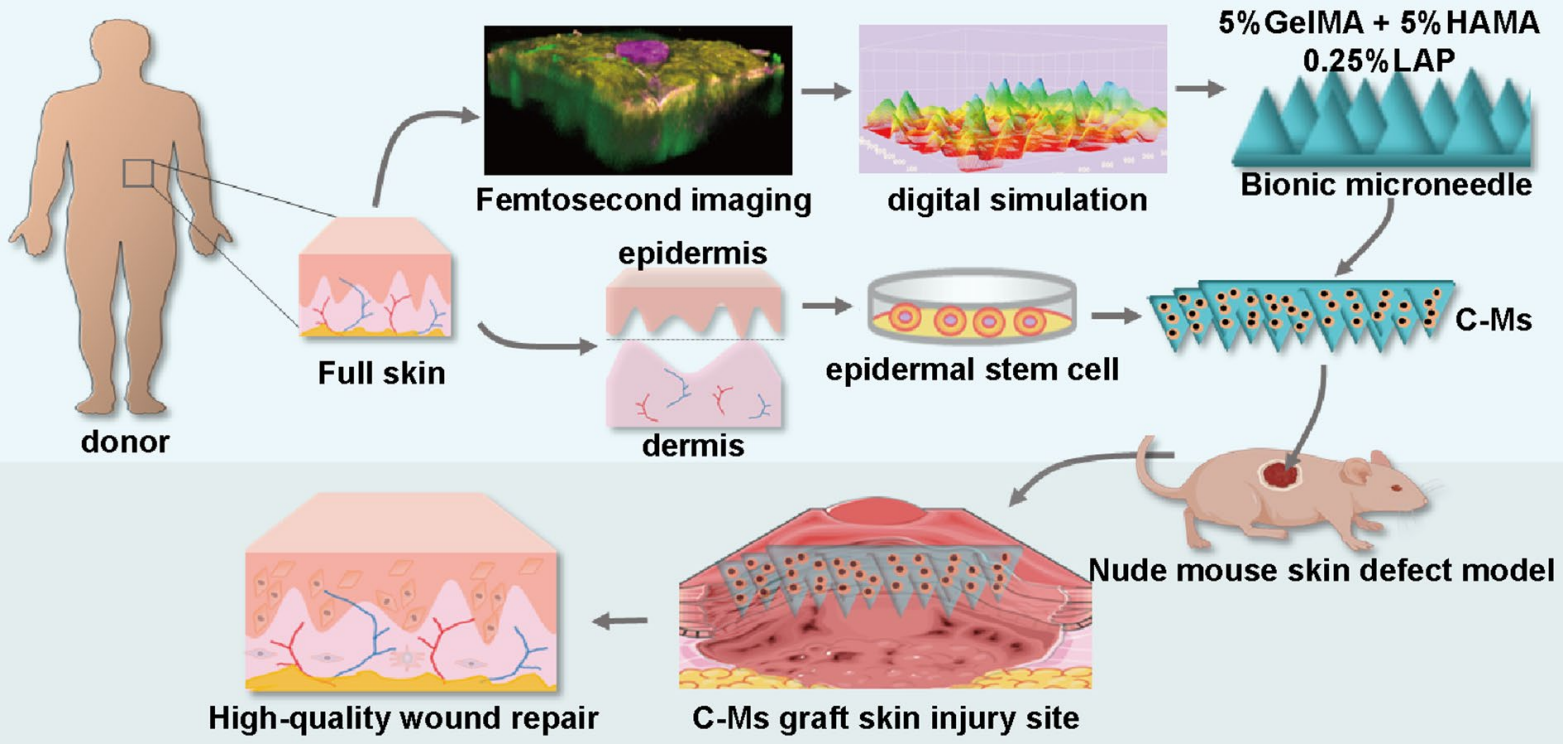

**Supplementary Information:**

The online version contains supplementary material available at 10.1186/s12951-025-03430-x.

## Introduction

Cell therapy has been a major research direction for replacing traditional skin grafting strategies after burns. The main treatment for large burn wounds is skin grafting, which causes iatrogenic injury to the donor site and is also limited by skin availability [1]. Skin stem cells are considered ideal seed cells for wound epithelialization due to their powerful self-renewal capacity and ability to generate highly differentiated progeny cells [2]. Previous studies have reported that keratinocytes derived from iPSCs or embryonic stem cells accelerate the healing of deep second-degree burn wounds in mice, but the potential tumorigenic effects hinder their clinical application [3]. Transplantation of autologous skin cells (such as epidermal cells or fibroblasts) or tissue-engineered skin substitutes (such as epidermal cell sheets or engineered composite skin) can address the issue of insufficient skin availability in patients [1]. However, subsequent clinical trials and follow-up found that the healed skin lacked the characteristic undulating microstructure at the dermal-epidermal junction, resulting in quality problems such as poor mechanical properties of the newborn skin, easy rupture, and blisters [1, 4]. Although our previous research has shortened the in vitro expansion period of epidermal stem cells (EpiSCs) and optimized the cellular therapy approach for burns, we have not been able to overcome the bottleneck of reconstructing skin anatomy in clinical trials.

The inability of wound cell therapy to reconstruct skin anatomy contributes to suboptimal wound repair outcomes [5]. Histopathologic studies have shown that minor human skin wounds heal effectively without intervention. In contrast, healing of large skin defects (burns) often results in regenerated skin lacking rete ridges (RRs), which in turn leads to impaired quality of the healed skin [6]. RRs are the specific niche of the EpiSCs, which regulate stem cell development and maintain skin homeostasis [7]. The undulating structure of the RRs enlarges the area of the dermal-epidermal junction while increasing the density of hemidesmosome along the basement membrane (BM). This three-dimensional structure enhances the anchoring stability of EpiSCs through hemidesmosomes, enabling them to respond to extracellular matrix (ECM)-mediated mechanochemical signaling. The dermis demonstrates extensive vascularization, coordinating dual roles: sustaining epidermal metabolic demands via nutrient perfusion and mediating interlaminar communication through paracrine signaling cascades, thereby driving ECM structural reorganization [8]. Pathological conditions like epidermolysis bullosa are marked by complete RR absence, whereas psoriasis features aberrant RR architecture [9]. Additionally, excessive friction, prolonged UV exposure, and aging can lead to abnormal RR structure [10]. Currently, researchers have reviewed the reconstruction strategies for RRs [11]. For example, collagen has been used to fabricate tissue-engineered skin in vitro by using RR molds, but the reconstructed RRs after in vivo transplantation exhibited poor fidelity. A composite hydrogel of GelMA-PEGDA-RGD was employed to create an RR model via projection micro-stereolithography, and the results demonstrated that the composite hydrogel promotes the formation of RRs and reconstructs the EpiSC niche. Soejima et al. found that the combination of human adipose-derived stem cells (hASCs) with artificial dermis enhances the formation of the BM. Although biomimetic technologies and the regulation of stem cell functions have shown promising prospects in reconstructing the microstructures of RRs, the complete reconstruction of RRs remains challenging. Therefore, as the main research direction of wound repair, cell therapy has important research value and far-reaching clinical significance in reconstructing the RRs.

Recently, microneedle-mediated cell therapy has emerged as a promising strategy, providing a new approach for RRs reconstruction. Microneedle (MN) arrays target penetration into the dermal reticular layer and localized delivery of drugs and cells, concurrently modulating tissue-level biomechanical properties and ultrastructural organization via mechanical signaling pathways involving integrin-FAK signaling [12]. Our team previously found that MNs inhibit the formation of scars in the skin through mechanobiological modulation via YAP/TAZ signaling [13]. Notably, CAR-T cell-conjugated porous MN scaffolds attenuated B16-F10 melanoma progression through sustained local IL-2 secretion [14]. Micropatterned MN delivering Foxp3 + Tregs reduced psoriatic plaque thickness via IL-10/TGF-β1 axis [15]. MN patches encapsulating umbilical cord MSCs accelerated re-epithelialization through Wnt/β-catenin activation [16], and thermo-responsive MN matrices loaded with ADSC spheroids enhanced diabetic ulcer perfusion via VEGF-α/ANG-2 paracrine cascades [17]. While previous studies have investigated the significant advantages of cryomicroneedles, stem cell-integrated MNs, and cell sheets for cell delivery in regenerative therapies, there have been fewer studies using specific morphologies of bionic MNs to remodel cellular niches by physically occupying them at target tissue sites [18, 19]. The planar anatomical structure of RRs closely resembles the shape of MNs. Therefore, we hypothesize that providing a biomimetic RR structure in skin defects could offer topographical clues for cells, thereby facilitating RRs regeneration. Previous studies have shown that anatomical structure and mechanical mechanics mediate collagen deposition and scar formation [11]. However, complete RR reconstruction remains challenging. During early embryonic development, basal stem cells in the oral mucosa induce the formation of RRs by promoting cell proliferation through mechano-chemical signals, generating intercellular forces [20]. In conclusion, we conjecture that the biomimetic MNs system loaded with EpiSCs may be feasible for the reconstruction of RRs.

**Scheme 1:**
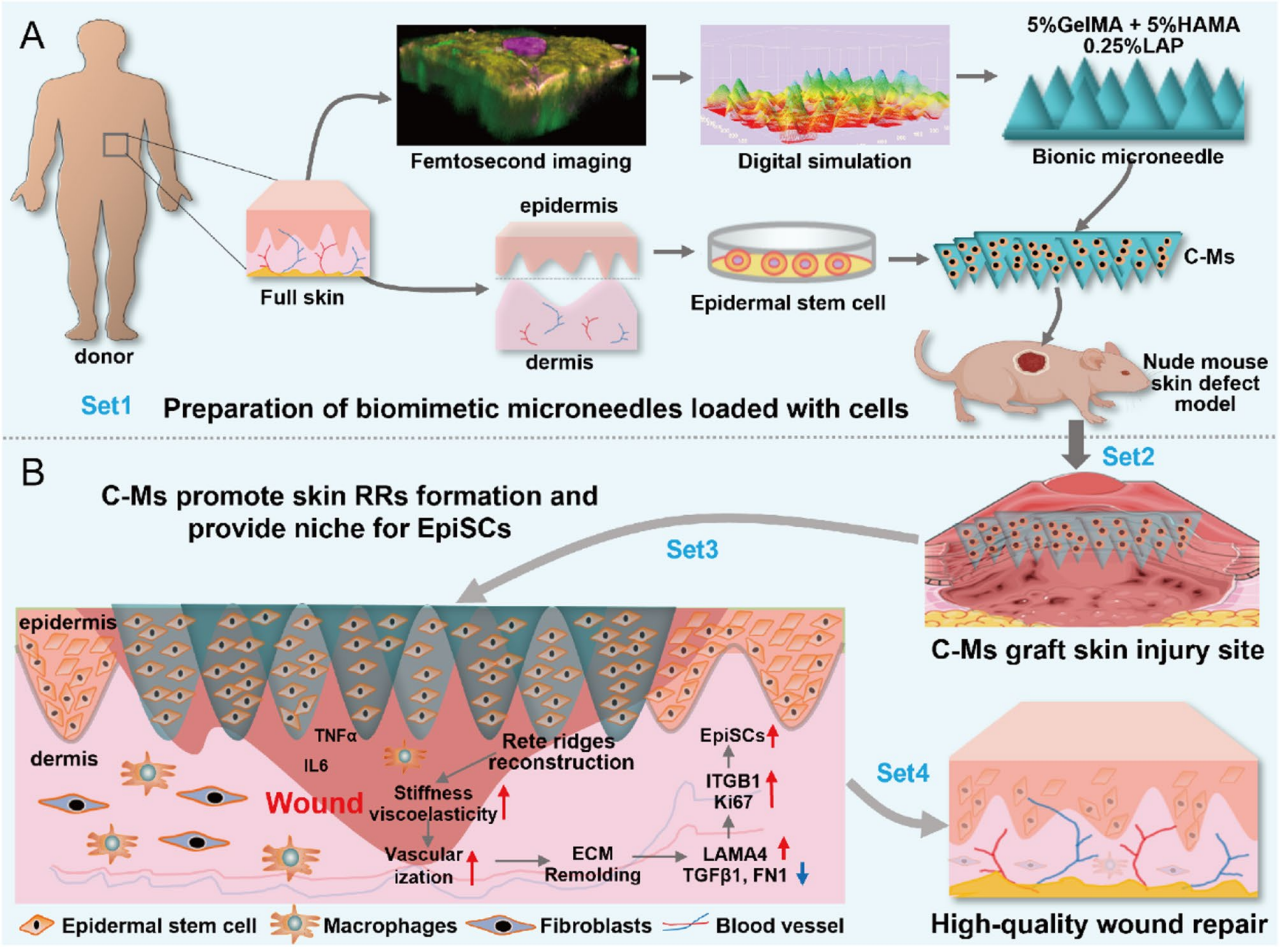
Diagram of C-Ms preparation and application. (**A**) Schematic of the fabrication of C-Ms. (**B**) The application and mechanisms of the C-Ms during in situ rete ridges reconstruction

This study aims to reconstruct the RR structure in wound healing through utilizing C-Ms, building upon our previous research (Scheme 1). The goal is to restore the RR structure through the physical placement of MNs directly into the wound. First, the RR structure of normal skin was analyzed and digitally simulated using femtosecond laser 3D reconstruction and serial tissue sectioning techniques. Biomimetic MNs were then fabricated using GelMA and HAMA materials through a demolding process. The safety of the cell-loaded biomimetic MNs was validated in vitro. Finally, in a nude mouse skin defect model, C-Ms successfully achieved RR reconstruction. Preliminary finding suggests that this might be related to C-Ms reshaping the ECM and providing a niche for EpiSCs. This study provides a simple and feasible approach for wound therapy with autologous epidermal cells by reconstructing RRs and stem cell niches, ultimately improving the wound healing quality.

## Results

### Observation and modeling of human RRs structure

**Fig. 1:**
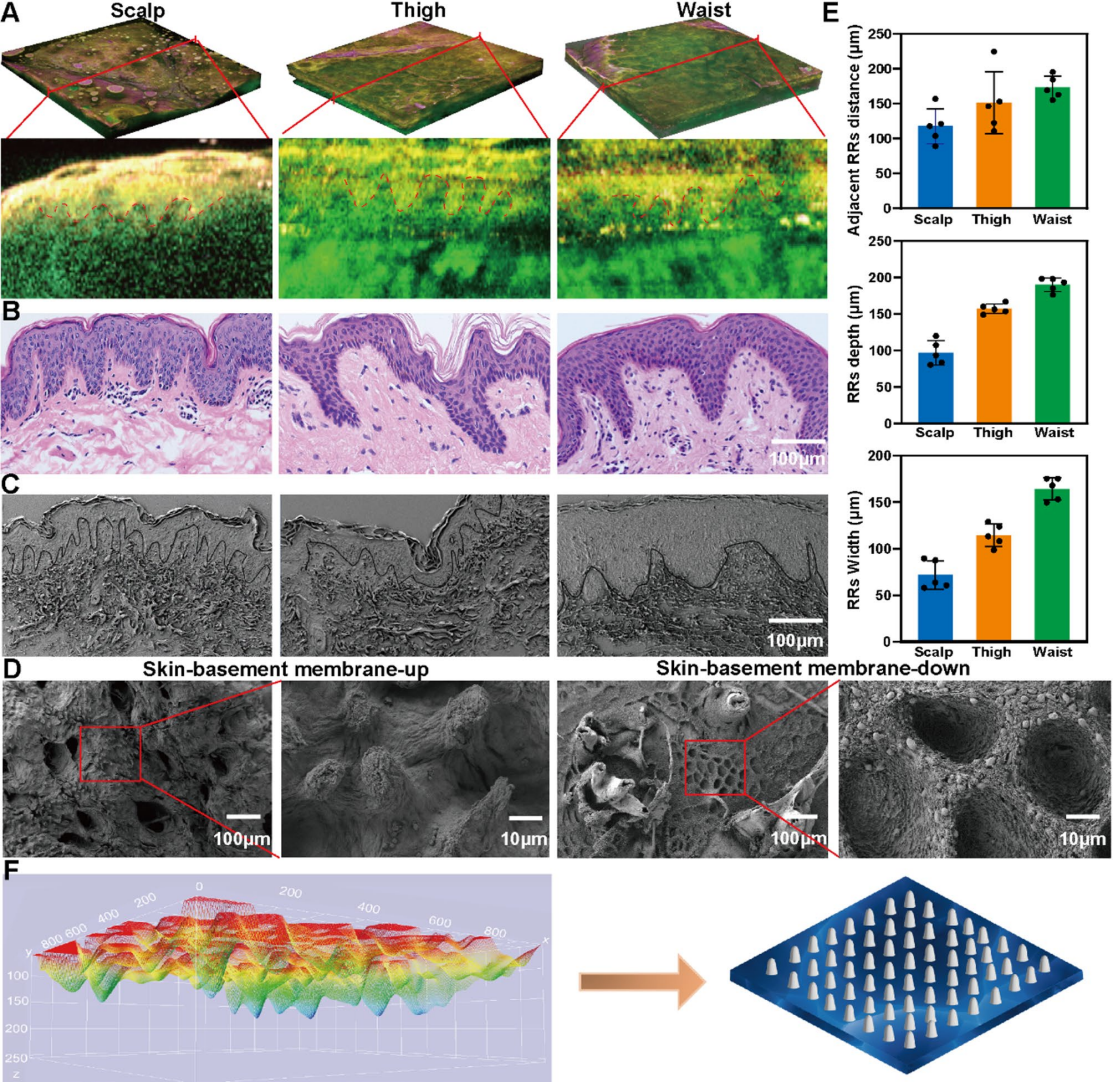
Native parameters of RR structure of human skin. (**A**) Laser femtosecond 3D imaging of different skin samples. (**B**) HE staining of different skin samples. (**C**) SEM results of different skin samples. (**D**) SEM results of the contact site of waist epidermis and dermis. (**E**) Parameters of RRs structure at different sites, including spacing, width and depth. (**F**) 3D digital modeling of the RRs structure of waist skin

In order to biomimetically construct the skin RRs structure, with the approval of the ethics committee and the consent of the volunteers (burn patients), full-thickness skin tissue was obtained to observe and digitally model the structure of the skin RRs. Femtosecond laser pulse microscopic 3D imaging technology, SEM and HE staining are effective methods to observe the structure of skin samples. The 3D imaging results of the skin were shown in Fig. 1A, and the skin RR structure was clear in the BM layer. To obtain native data on the RR structure, skin samples were serially sectioned (6 μm). The results of HE staining (Fig. 1B) and SEM (Fig. 1C) and parameters of RRs structure (Fig. 1E) showed that there were significant differences in the spacing, width and depth of the RR structures at different sites. The scalp presents densely stacked stratum corneum, while the thigh skin and the waist skin present smoother, larger stratum corneum. In addition, we separated the epidermis and dermis layers and observed them by SEM (Fig. 1D). The junction sites between the epidermis and dermis layers had dense collagen structures, and the hemidesmosome structure was obvious. There were honeycomb-like holes in the upper dermis layer of the BM, and basal cells were stably embedded in the ECM through hemidesmosomes. And the hair follicles penetrate the dermis and epidermis layers. Finally, we performed 3D digital modeling of the RRs structure (Fig. 1F and S1) and found that the RR structure was similar to matrix MNs. It has been reported that the shape and density of the RRs change with age, mature in adulthood, and then gradually shrink [21, 22]. Therefore, in this study, we selected the RR parameters of the 21-year-old waist skin to simulate the biomimetic RR matrix MNs.

### Preparation and physical properties of biomimetic RR microneedles

**Fig. 2:**
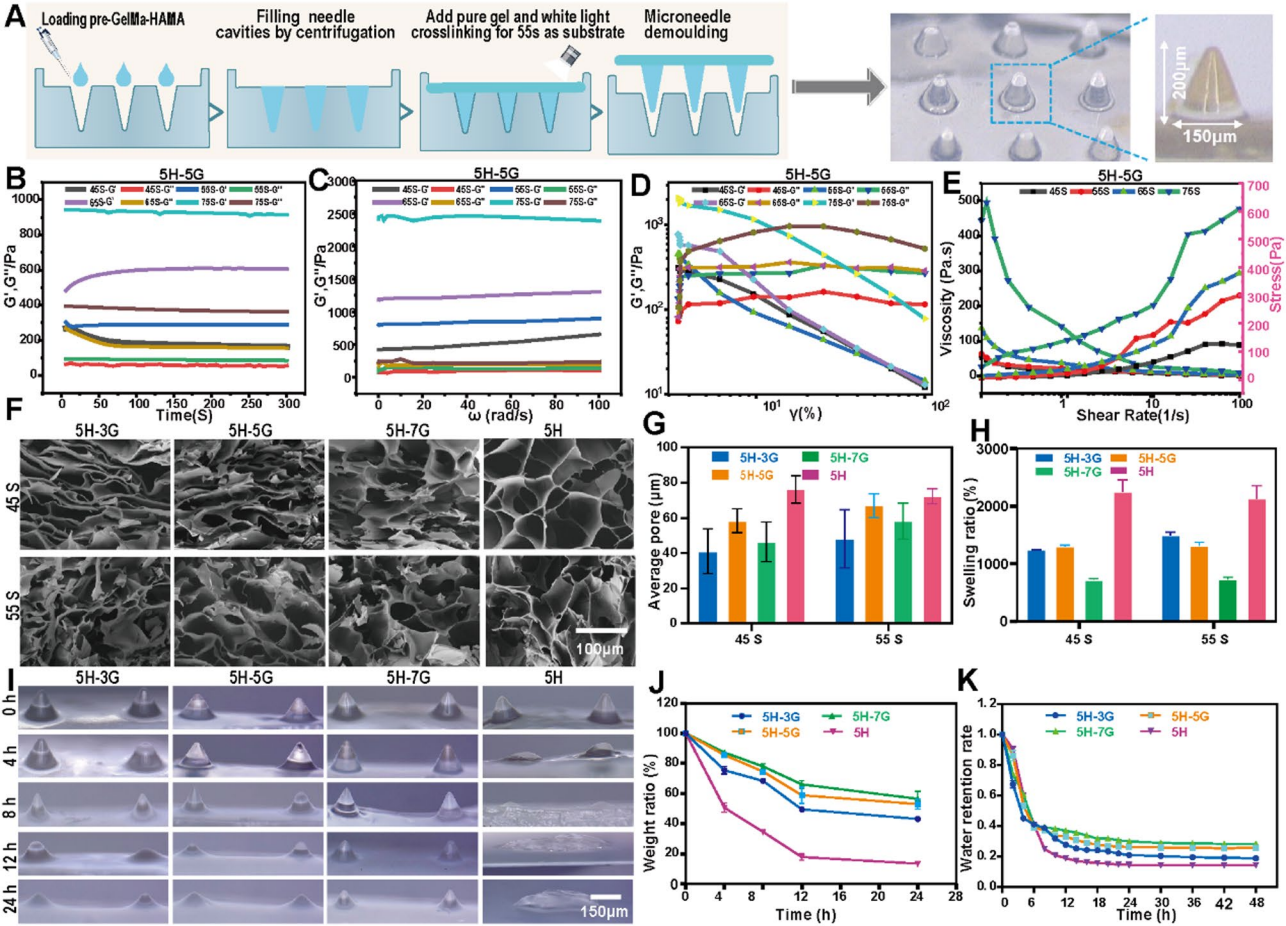
Evaluated the physical properties of MNs. (**A**) Preparation process and size of MNs. (**B**) Oscillation time sweep curves related to 5H-5G. (**C**) Oscillation frequency sweep curves related to 5H-5G. (**D**) Oscillation strain sweep curves related to 5H-5G. (**E**) Flow frequency sweep curves related to 5H-5G. (**F**) SEM results of MNs at 45 and 55 s. (**G**) Statistical distribution of MN gap size at 45 and 55 s. (**H**) Swelling properties of cross-linked time of 45 and 55 s MNs after 24 h. (**I** and **J**) Degradation of cross-linked time of 55 s MNs in type II collagenase solution in vitro. (**K**) Water retention properties of MNs at 55 s

Gel and HA are natural bioactive components of ECM, with excellent biocompatibility, and have good application prospects in tissue engineering, cosmetology and other fields [23]. We have also found that hydrogels prepared by GelMA and HAMA exhibit excellent biomechanical properties in tissue adhesion, wound healing, and hemostasis [24]. In this study, Gel and HA were used to synthesize GelMA and HAMA respectively to prepare bionic RR MNs. In order to quickly prepare the imitation skin RR MNs, the MN mold was prepared by PDMS, and then the MN patch was formed by solution pouring. The bottom diameter of the formed MNs was 150 µm, the height was 200 µm, and the MN spacing was 200 µm (Fig. 2A). Then we researched the effect of HAMA and GelMA mixed at different final mass ratios (5%HAMA + 3%GelMA, 5H-3G; 5%HAMA + 5%GelMA, 5H-5G; 5%HAMA + 7%GelMA, 5H-7G; 5%HAMA, 5H) and crosslinking time (25, 35, 45, 55, 65, 75 s) on microneedle molding. As shown in Figure S2E, MNs photo-crosslinked at 25 and 35 s could not be stably formed, and the morphology of MNs was intact at 45, 55, 65 and 75 s. Therefore, these crosslinking times of 45, 55, 65 and 75 s were selected to continue studying the physical properties of biomimetic MNs. For further characterization of MNs, we analyzed the elastic modulus (G’) and the viscous modulus (G’’) as a function of angular frequency (ω), oscillation time, oscillation strain and flow frequency at 25 ºC. In the oscillation time and frequency sweep curves (Fig. 2B-C, S2A-B), progressive enhancement in G’ and G” was observed with prolonged crosslinking durations and elevated GelMA concentrations, exhibiting a dominant elastic solid behavior (G’ > G”), which supports long-term cellular encapsulation. In the amplitude scanning analysis (Fig. 2D, S2C), a narrow linear viscoelastic region (strain ≤ 10%) was found in all groups, indicating a brittle hydrogel. Notably, GelMA incorporation improved network resilience through secondary covalent interactions. Flow sweep curves (Fig. 2E, S2D) demonstrated that hydrogels photo-crosslinked at 45 and 55s maintained shear stresses below 200 Pa under physiological shear rates (0.1–10 s⁻¹), ensuring encapsulated cells could potentially be protected from mechanical damage. Given that the C-M in this study was intended to be used for granulation tissue after large skin defects, the mechanical strength requirements for MN were relatively low, and we only needed to ensure that the cross-linking time was sufficient for the efficacy and functionality of cell delivery. Therefore, we selected 45 and 55 s as the optimal cross-linking times for subsequent studies. SEM observation of the surface morphology of the MNs matrix (Fig. 2F) found that the surface of the MNs of 5H-3G, 5H-5G, and 5H-7G after cross-linking for 45 and 55 s was smooth and had dense and interconnected porous structures. ImageJ counted the pore sizes of each group (Fig. 2G), and the 5  H had a larger aperture structure. In addition, the moisture balance of the wound will affect wound healing, so the water-absorbing and water-retaining properties of the MNs were further tested. The swelling performance results of MNs showed that at 45 and 55 s (Fig. 2H), the swelling rate of 5% HAMA alone was the highest, and the lowest in the 5H-7G group. There was no difference in the swelling ratio (55 s) among all groups compared with that at 45 s. For the water-retaining capacity of MNs (Fig. 2K and S2F), in all groups photo-crosslinked for 45 s, the water retention capacity was lower compared with that at 55 s. Finally, the MNs were treated with 2.5 U/ml collagenase type II solution in vitro to simulate the in vivo degradation rate (Fig. 2I and J and S2G), and the degradation rates of 5H-3G, 5H-5G, 5H-7G and 5H after gelation within 55 s were slower than those after gelation within 45 s. In summary, the MNs formed by 5H-5G at 55 s of photo-crosslinking have complete moldability, appropriate pore size, storage modulus and degradation speed, and have good swelling ratio and water retention properties. Therefore, the bionic RR MNs formed by 5H-5G at an optical crosslinking time of 55 s were adopted in the subsequent study.

### Biosafety of bionic skin RR MNs

**Fig. 3:**
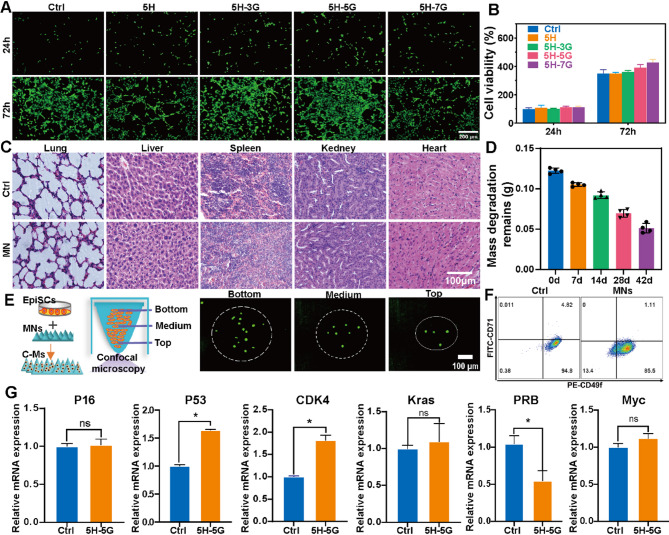
Evaluated the biosafety of bionic RR MNs. (**A**) Live cell staining of 3T3 cells treated by RRs extract. (**B**) CCK8 results of 3T3 cells treated by RRs extract. (**C**) HE staining results of the heart, liver, spleen, lung, kidney. (**D**) Degradation of MNs at different time points after subcutaneous transplantation. (**E**) At 24 h, the survival of EpiSCs in MNs was observed using a confocal laser microscope. (**F**) At 24 h, the expression of CD71^−^CD49f^+^ in EpiSCs in C-Ms and the control group was detected by flow cytometry. (**G**) The expression of representative genes of EpiSCs was detected by qPCR. (*P < 0.05)

Biomimetic RR MNs need to have good biological safety for treating wounds. First, we prepared 5H, 5H-3G, 5H-5G, and 5H-7G into extracts in DMEM medium containing 10% FBS, and then evaluated the toxicity of the extracts on mouse 3T3 fibroblasts. The results of living cell staining (Fig. 3A) and CCK8 (Fig. 3B) test showed that there was no significant difference in the number of living cells in each extract group compared with the control at 72 h, and the cell activity was good. Then 1 cm by 1 cm matrix MNs (5H-5G) of approximately 120 mg were transplanted subcutaneously under the skin of the back of C57 mice to evaluate in vivo degradation rate and inflammatory infiltration. By 42 days, the degradation rate of MNs was approximately 60% (Fig. 3D). Compared with the control group, the inflammatory infiltration of the subcutaneous transplantation site of MNs was not obvious and the inflammatory factors (IL-1β, TNF-α) basically returned to the normal levels (Fig. S3A-B), and there was no pathological injury of the organs (heart, liver, spleen, lung, kidney) in the two groups (Fig. 3C). In addition, no hemolysis occurred in the microneedle extract (Fig. S3C).

The RR MNs designed in this study need to deliver EpiSCs, so we further evaluated the ability of the RR MNs to load EpiSCs. C-Ms were prepared by mixing 1.6 × 10^6 EpiSCs with 1 ml MN solution, and were cultured conventionally. After 24 h, the survival of EpiSCs was observed by living cell staining, the expression of EpiSCs specific markers (CD71 and CD49f) was detected by flow cytometry, and the expression of representative genes of EpiSCs proliferation, senescence and tumorigenesis was detected by qPCR. As shown in the staining results of living cells (Fig. 3E), living cells were present at the bottom, middle and top of the MNs. The proportion of living cells remained above 90% after 24 h (Fig. S3D). There was no statistical difference in the proportions of CD71^−^ and CD49f^+^ between the C-Ms and control (Fig. 3F). The effects of MNs on EpiSCs gene expression showed that compared with control (Fig. 3G), the expressions of P53 and CDK4 in MN group was increased (*P* < 0.05), while the expressions of PRB were decreased (*P* < 0.05), and the expressions of P16, Kras and MYC were not significantly different between the two groups. The above results indicate that C-Ms have good biosafety and can be used for further studies.

### C-Ms accelerate wound healing in mice through rapid vascularization and promote skin RRs regeneration

**Fig. 4:**
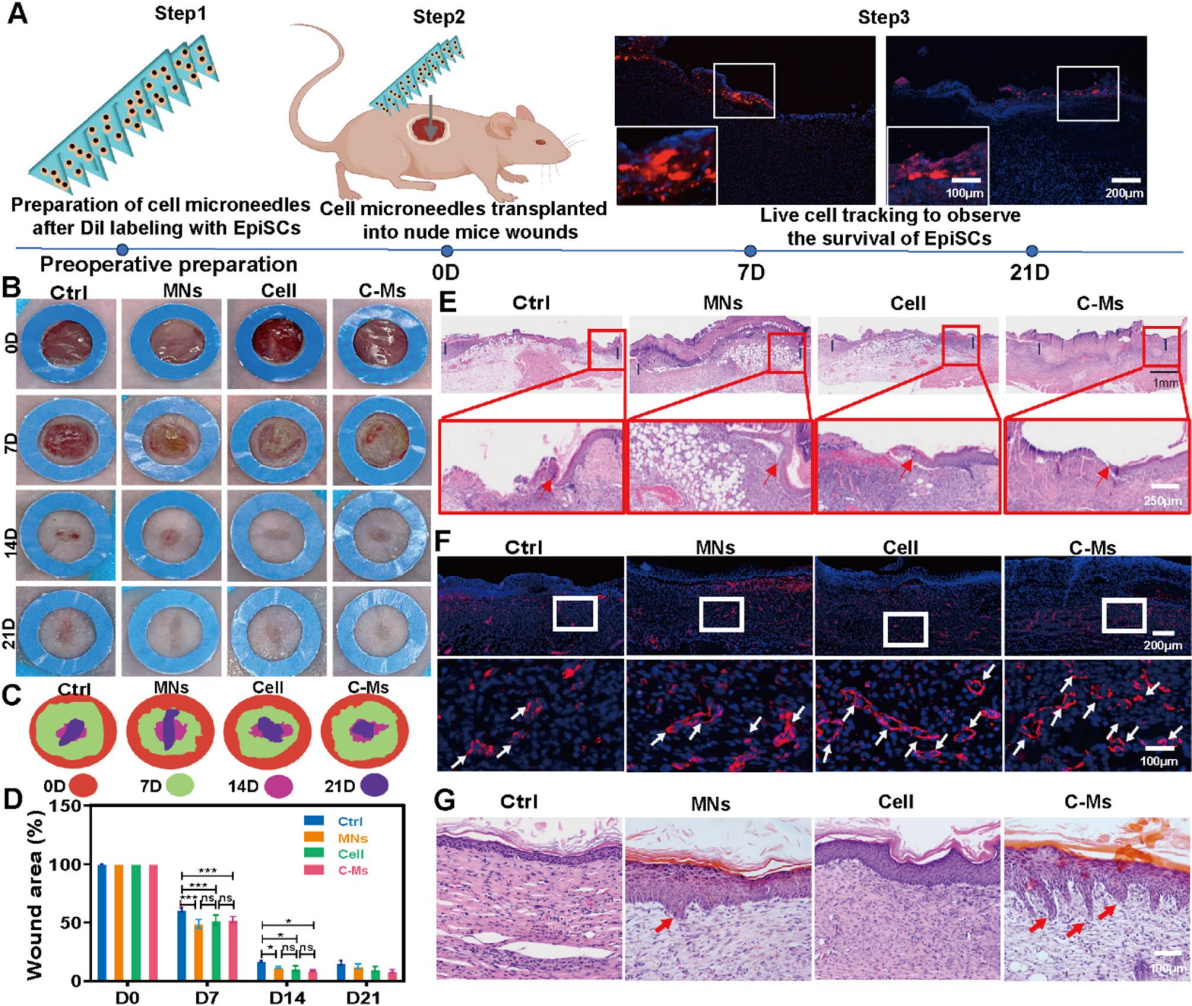
C-Ms accelerate wound healing, rapid vascularization, and promote skin RRs regeneration. (**A**) Living cells tracked the survival of transplanted EpiSCs at different times after C-Ms were transplanted to the wound. EpiSCs were marked with Dil dye. The diameter of the full-thickness skin defect wound in nude mice was 1 cm. (**B**) Digital camera photographed the wounds in each group at different time points. The inner diameter of the blue circle was 1 cm. (**C** and **D**) Statistical results of unclosed wound area in each group at different time points. (**E**) HE staining results on the 7th day of each group. The blue line, the red box, and the red arrow indicated the location of epithelialization. (**F**) On day 7, showed Alexa Fluor^®^ 555 anti-mouse CD31 immunostaining results of wounds in each group. White boxes and white arrows represented new blood vessels. (**G**) On day 21, HE staining results in each group. The red arrow indicated human-like new RRs

To observe the survival of EpiSCs after biomimetic EpiSC-microneedles (C-Ms) implantation in the wound, we labeled EpiSCs with Dil and prepared them into C-Ms. And then, C-M was transplanted into a 1 cm by 1 cm full-thickness skin wound in nude mice and was followed up for 21 days (Fig. 4A). Wound samples were collected for cryosections on postoperative day 7 and day 21. Laser confocal results showed that implanted EpiSCs were observed in the BM layer of the skin, but the number of surviving EpiSCs decreased by day 21. After that, C-Ms without Dil label were used for follow-up experiments. Controls included an untreated wound, wounds treated with only cells and MNs respectively. Wound closure in each group was recorded by digital camera (Fig. 4B), and wound unclosed area was measured by planimetry (Fig. 4C and D). By day 7, the wound unclosed area in the MNs, cell and C-Ms groups was significantly larger than that in the Ctrl (*P* < 0.001), and there was no significant difference among the MNs, cell and C-M groups. By day 14, the wounds of in the cell, MNs and C-M group was completely closed. By day 21, the wound in the Ctrl was completely closed. HE staining at day 7 and day 21 confirmed the closure efficiency of C-M treated wounds (Fig. 4E and G). By day 7, the un-epithelialization in the MNs, cell and C-Ms groups was significantly greater than that in the control group (Fig. S4A, *P* < 0.001). By day 21, wound epithelialization was completed in each group, but the epidermal barrier function of the wound in the C-M treatment group was the most obvious, and the new epidermis was thicker and had a multi-layer epidermal structure, which was close to that of normal skin (Fig. S4B).

Early neovascularization provides necessary nutrients for wound healing, accelerates wound repair and inhibits scar formation, so vascularization is a key factor in evaluating skin wound healing [25]. Capillaries were immune-stained with CD31 to quantify vascularity of the healing wounds on days 7 and 14. By day 7 (Fig. 4F and S4D), C-M treated wounds showed higher blood vessel density (67.3 ± 4.1) compared to the Ctrl, cell and MN groups (25.3 ± 2.1, 49.3 ± 4.9, 33.3 ± 3.3; *P* < 0.01). By day 14 (Fig. S4C and S4E), after the wound was completely healed, the blood vessel density of the C-M treatment group (19.3 ± 2.8) gradually returned to normal levels compared with the Ctrl, cell and MN groups (46.3 ± 4.4, 33.0 ± 3.7, 27.6 ± 2.4; *P* < 0.01). The C-M treated wounds formed human-like RRs by day 21 (Fig. 4G), and the MN group alone also had part of RRs formation, while the epidermis of the Ctrl and cell groups was flat and had no RRs formation.

### C-Ms promotes skin RRs formation and provides niche for episcs

**Fig. 5:**
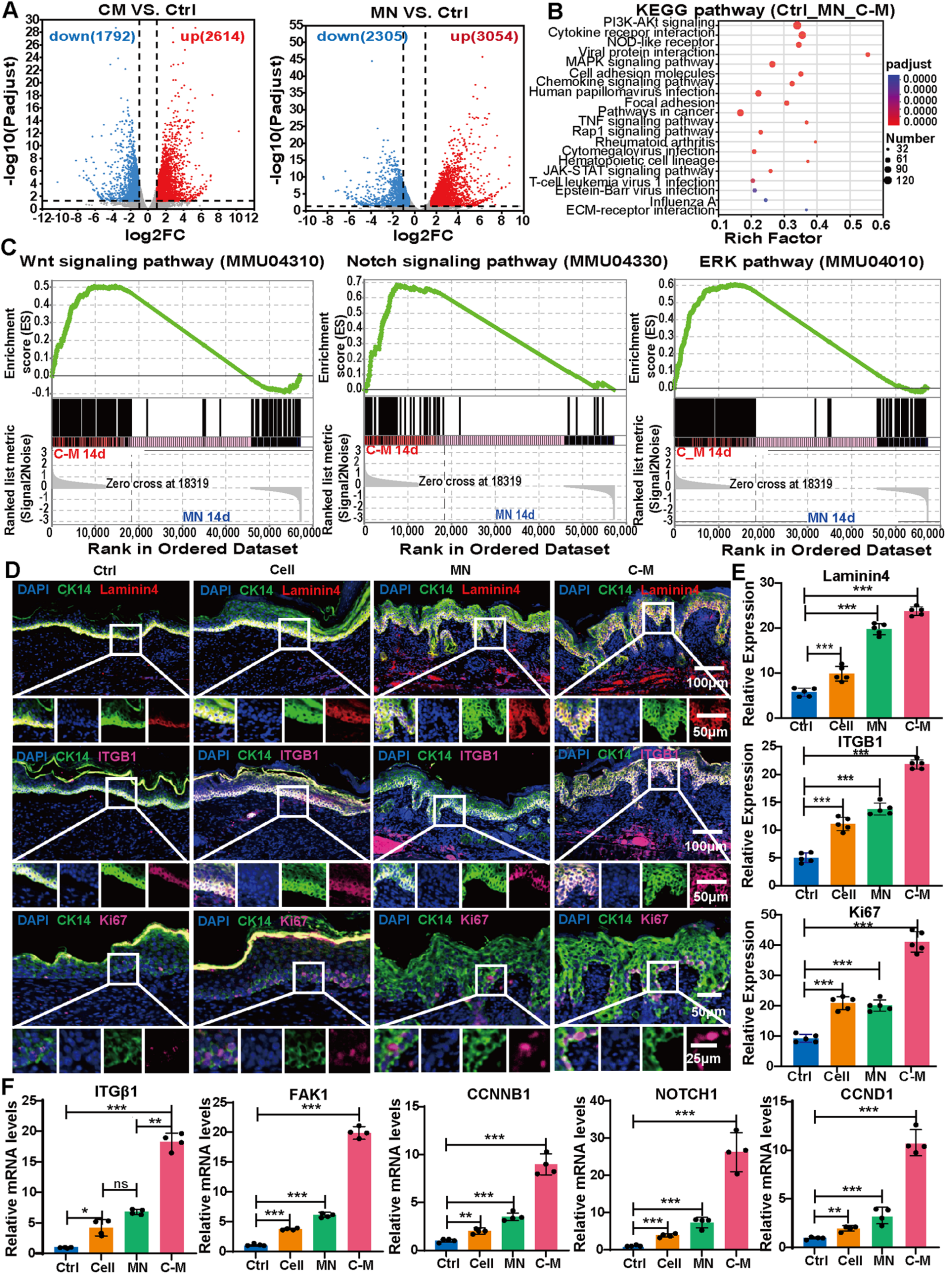
C-Ms promotes skin RRs formation and provides niche for EpiSCs. (**A**) The volcano map shows up-regulated and down-regulated genes identified 14 days after treatment with MN and treatment with C-M. (FC ≥ 2.0, *P* < 0.05). *n* = 3. (**B**) KEGG analysis of genes significantly changed in the gene set 3. (FC ≥ 2.0, *P* < 0.05). (**C**) Gene set enrichment analysis (GSEA) of RNA-seq data for C-M vs. MN. (**D**) Representative immunofluorescent staining of control, cell only, MN only and C-M only 21 days after transplantation. Top row: Anti-mouse laminin4 (red), DAPI (blue) and anti-CK14 (green). Middle row: Anti-mouse ITGB1 (pink), DAPI (blue), and anti-CK14 (green). Bottom row: Anti-mouse Ki67 (pink), DAPI (blue), and anti-CK14 (green). (**E**) shows the statistical result of the fluorescence Fig. 5D. (**F**) The expression of representative genes of skin tissues (day 21) was detected by qPCR. (*P < 0.05)

In order to elucidate the mechanism of C-Ms promoting the regeneration of human-like RRs structure on the wound of nude mice, healed wound samples were collected 14 days after surgery for RNA sequencing analysis. Studies have reported that mechanical MN molds are used to simulate RRs in vitro and then transplanted into wounds to reconstruct RRs with poor fidelity [26]. Therefore, we further focused on the influence of MN on RRs formation. Differential gene statistics were shown below (Fig. 5A), compared with the Ctrl group, there were 3054 up-regulated genes and 2305 down-regulated genes in the MN group; and there were 2614 up-regulated genes and 1792 down-regulated genes in the C-M group. However, there was no significant change in the number of differential genes in the MN group compared with the C-M group (Fig. S5A). Using Wayne diagram displaying of the differential genes in the Ctrl, MN and C-M groups, we found that 1697 differential genes (gene set 1) were only in the MN vs. the Ctrl, 744 (gene set 2) were only in the C-M vs. the Ctrl, and 3662 (gene set 3) were in the intersection (Fig. S5B). Then, KEGG enrichment analysis of the gene set 3 found that focal adhesion kinase (FAK), ECM receptor interaction, and other pathways were significantly enriched, which were related to the differential genes LAMA4, ITGB1 and FN1 (Fig. 5B and S5C and S5D). ECM provides anchoring sites for basal cells. Upon binding to ECM components such as laminin and collagen via integrins, FAK is activated, triggering downstream signaling pathways that regulate the polarization and migration of basal cells, thereby promoting the morphogenesis of the reticulate ridge [27, 28]. qPCR analysis of ITGB1, FAK1, COL1A1, and LAMA4 revealed significant activation of ECM receptor interaction and FAK in the C-Ms group (Fig. 5F and S5F). Moreover, LAMA4 is the main regulatory molecules involved in the formation of BMs [29]. Therefore, we performed immunofluorescent staining of LAMA4 on the wounds 14 and 21 days after surgery. On day 14 (Fig. S6) and day 21 (Fig. 5D and E), the expression of LAMA4 in the C-M treated group was significantly higher than those in the MN and the Ctrl (*P* < 0.001), and the MN group was significantly higher than the Ctrl (*P* < 0.05).

Further GO enrichment involved the signaling pathway for the EpiSCs maintenance, and the results revealed characteristic changes related to stem cell proliferation, cell adhesion, and epidermal cell differentiation (Fig. S5E). GSEA analysis showed that Notch, Wnt and ERK-related signaling pathways were significantly affected after C-M treatment compared with MN (Fig. 5C). Studies have reported that the loss of Notch signaling leads to premature differentiation of basal cells, disrupting epidermal stratification and the structure of RRs [30]. Wnt/β-catenin signaling promotes ECM remodeling at the dermal-epidermal junction by regulating MMP9 expression. The absence of ERK activity results in reduced proliferation of epidermal basal cells and disorganization of RRs structure [31]. Based on qPCR analysis of NOTCH1, Ctnnb1, Axin2, and Ccnd1, we found that Notch, Wnt/β-catenin, and ERK pathways were significantly activated in the C-Ms group (Fig. 5F and S5F). In order to intuitively study stem cell maintenance, we analyzed the expression of epidermal cell proliferation marker Ki67 and stem cell marker ITGB1 in each group. The results showed that Ki67 and ITGB1 were more abundantly expressed in the C-M treatment (Fig. 5D and E). In general, C-M treatment is more conducive to providing a niche for stem cells.

### C-Ms improve wound healing quality in mice by remodeling extracellular matrix

**Fig. 6:**
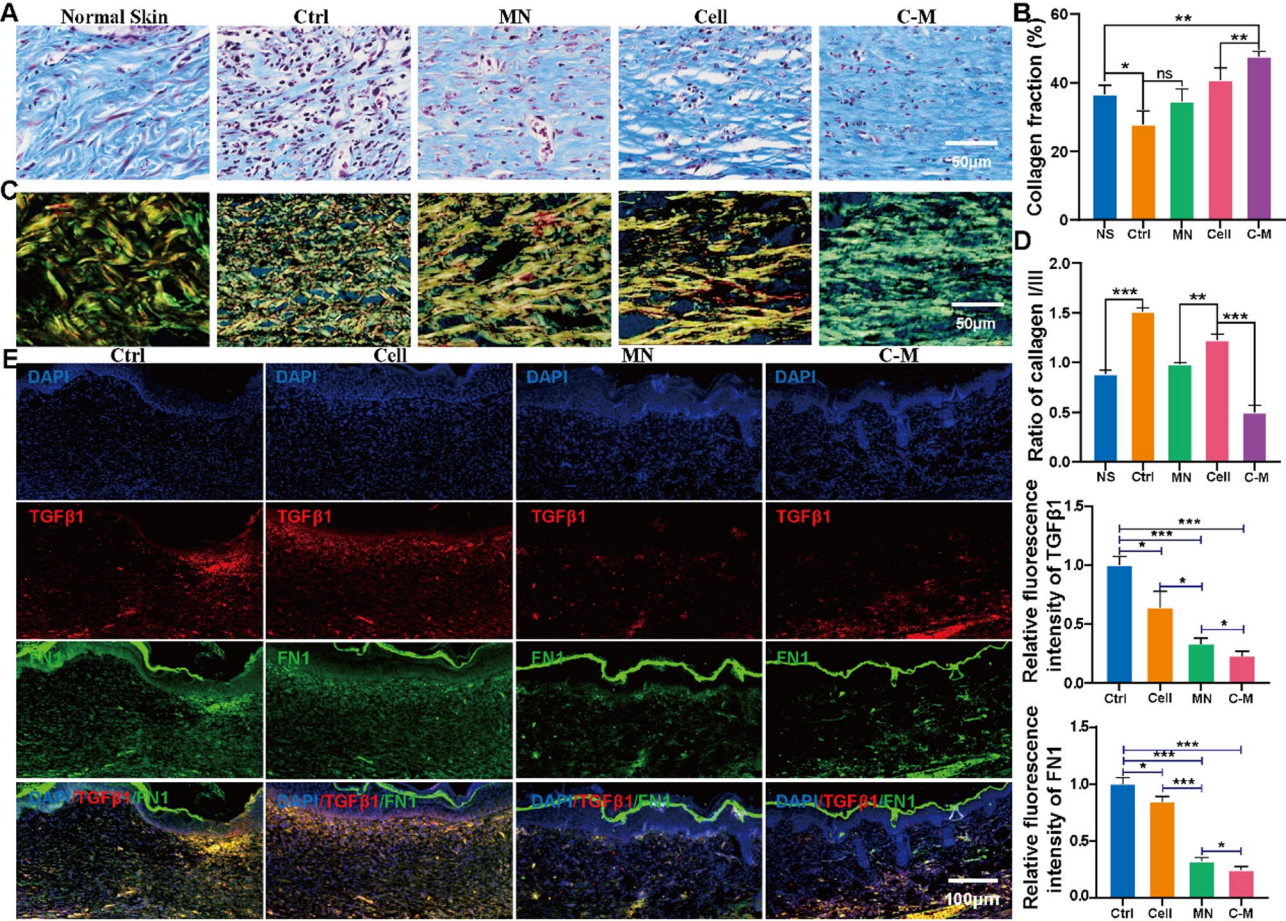
C-Ms remodeled the extracellular matrix. (**A**) Masson’s staining of the C-Ms group, MN group, Cell only group and wound only group at day 21. (**B**) It was the statistical result of A. (*P < 0.05). (**C**) Picrosirius red staining of the C-Ms group, MN group, Cell only group and wound only group at day 21. (**D**) It was the statistical result of collagen I / III of C. (*P < 0.05). (**E**) Representative immunofluorescent staining of control, cell only, MN only and C-M only 21 days after transplantation. Anti-mouse TGF β1 (red), DAPI (blue) and anti-mouse FN1 (green). (**F** and **G**) were the relative fluorescence statistics of TGFβ1 and FN1 of E. (*P < 0.05)

ECM remodeling is important for normal wound healing and reduced fibrosis. Masson staining was used to determine total collagen expression in regenerated skin wounds; Picrosirius red staining further determined the expression of type I and III collagen and collagen architecture. By day 21, Masson results were as follows (Fig. 6A and B); the total collagen fraction in the C-M group (36.7 ± 2.1%) was significantly higher than that in the Ctrl, cell and MN groups (27.9 ± 3.1%, 40.7 ± 2.8%, 34.7 ± 2.8%, respectively; *P* < 0.01); Picrosirius red results were as follows (Fig. 6C and D); the proportion of type III collagen in the C-M treatment group (0.9 ± 0.0%) was significantly higher than that in the Ctrl, cell and MN groups (1.5 ± 0.0%, 1.2 ± 0.0%, 1.0 ± 0.0%, respectively; *P* < 0.01). Its blue fibrous tissue was similar to normal skin. In addition, the wound fibers treated in the MN, cell and C-M groups were thick, with parallel fiber orientations and increased fiber thickness, while the fibers in the Ctrl were disorganized and the fibers were smaller.

Our research team previously reported that MNs regulate the process of skin fibrosis through mechanical action and inhibit scar formation [13]. TGFβ1 and FN1 are classic marker genes of fibrosis, so we further measured the expression of TGFβ1 and FN1 in healed wounds. By day 21 (Fig. 6E, F and G), immunofluorescent staining analysis showed that the expression of TGFβ1 in wounds in the C-M treated group was significantly lower than that in the MNs group (*P* < 0.01). Compared with the group and control group, the expression of TGFβ1 decreased in the MN group (*P* < 0.01). In addition, the expression of FN1 in each group had a similar trend to that of TGFβ1. The expression trends of TGFβ1 and FN1 mRNA in the skin tissues (day 21) detected by qPCR were consistent with the results of immunofluorescence (Fig. S5F).

## Discussion

Although wound cell therapy has progressed from basic research to clinical applications, it has long been constrained by the bottleneck of poor healing quality, leading to a temporary halt in its development. We have been engaged in long-term research on EpiSCs and autologous skin cell-based wound therapies, previously completed systematic explorations of the safety and efficacy of EpiSCs [32]. In this study, we employed a simple, convenient, and cost-effective approach using C-Ms to reconstruct RRs in situ, accelerating wound vascularization, remodeling the ECM, and reducing fibrosis. This significantly improves the quality of wound healing and opens up new strategies for solving bottlenecks and clinical research in wound cell therapy.

MNs may improve the rate of wound healing by accelerating early angiogenesis. In this study, MNs with RR-mimicking structures were constructed in vitro using bionic technology, and then loaded with EpiSCs to preliminarily explore their physical properties and biosafety, to be suitable for in vivo transplantation and subsequent potential clinical trials. C-Ms were then transplanted into wounds (diameter: 0.8 cm) in nude mice to accelerate wound healing and achieve human-like RR regeneration. Compared with untreated wounds, wounds treated only with MNs or C-Ms showed an early increase in the number of capillaries, which may be attributed to the remodeling of dermal fiber arrangement by C-Ms through mechano-biomechanical pathways (e.g., Hippo-YAP signaling). It was shown that fiber alignment is a key factor in rapid angiogenesis [33], which is consistent with our Sirius Red staining results. During small wound healing, peritraumatic keratinocytes migrate to promote wound re-epithelialization. However, in large wounds, keratinocytes are farther away from the point of trauma, resulting in delayed healing. Provision of exogenous EpiSCs to nude mice wounds accelerated wound re-epithelialization and epidermal regeneration compared to controls. In addition, the C-Ms group had highly humanized RR formation. Notably, EpiSCs respond to external mechanical stress and subsequently activate ERK/MEK cascade signaling. MMP9 degrades the ECM at the dermal-epidermal junction, promoting epithelialization and RR formation [20, 34]. Therefore, C-Ms and EpiSCs may increase MMP9 secretion, allowing keratinocytes at the wound edge to migrate into the wound. In MN-only and controls, the persistence of MMP9 may prevent migrating keratinocytes from adhering to newly synthesized BM, resulting in delayed wound healing.

The RRs structure plays a crucial role in maintaining the functional balance of the epidermis-dermis, but it is unable to repair itself after extensive skin damage. A study reported that oral RR structures could be reconstructed using biomimetic oral mucosa equivalents, suggesting that physical methods to reconstruct RRs are feasible [35]. We observed that C-Ms promoted the formation of RRs by remodeling the ECM, whereas the number and structural integrity of RRs in the MNs-only group were significantly lower than those in the C-Ms group. This difference may stem from the interaction between MNs, EpiSCs, and fibroblasts. Traditional split-thickness and full-thickness skins with relatively intact skin structures such as hair follicles, sweat glands, sebaceous glands, and RRs have significantly better quality of wound repair when compared with tissue-engineered skin substitutes. However, the limited availability of donor skin remains a bottleneck. Although C-Ms can promote the formation of RRs and improve wound healing, they still fall short of autologous skin grafts. The aim of this study was to utilize C-Ms technology to reconstruct RRs and thus improve the quality of wound cell therapy. Transcriptome sequencing analysis showed that the physical occupancy provided by bionic MNs activated ECM receptor interactions, focal adhesion, and cell adhesion molecule signaling pathways. Overall collagen expression was relatively high in C-Ms treated group, while relatively low in control group. It has been reported that neocollagenesis promotes angiogenesis and granulation tissue formation and accelerates wound closure [36]. C-Ms modulate collagen structure, and MN-mediated remodeling of the ECM exhibits a different collagen fiber size and arrangement pattern from that of natural skin. This difference may be due to the mismatch between exogenous bionic microneedle degradation and endogenous rete ridge regeneration kinetics, as well as spatial and temporal differences in mechanotransduction signals in the local microenvironment. Therefore, long-term observation of the integrity and functional balance of the RR maintained by C-Ms is needed. In addition, the ratio of type I/III collagen was significantly lower in the MNs group than in the control and cellular groups (*p* < 0.05), suggesting that MNs have the potential to inhibit scar formation. Previous studies reported that MNs inhibited mechanically mediated fibrosis during wound healing in mice [13, 37]. TGF-β1, FN1 and POSTN were key factors in determining the degree of fibrosis [38]. After wound healing, TGF-β1 and FN1 expression were significantly reduced in the C-Ms group (*p* < 0.05). This suggests that C-Ms can promote tissue regeneration and reduce scar formation, providing a new approach to improve the quality of wound healing.

Wound healing with cell therapy or traditional skin grafting often lacks RR structures in the regenerated skin, which severely impairs the biological functions of EpiSCs. On the one hand, RR formation results from the continuous proliferation and differentiation of basal stem cells. On the other hand, the undulating structure of RRs provides a niche for EpiSCs [20, 39]. Compared with the EpiSCs-only transplantation group, while both EpiSCs and C-Ms groups accelerated early wound healing, only the C-Ms group was able to form RRs. It is well-known that the stem cell microenvironment, including geometric and mechanical properties, influences cell proliferation and differentiation [40]. We observed a decrease in the survival rate of transplanted EpiSCs by day 21, which may be related to the viability of the transplanted cells, as cells with lower viability might die after transplantation. Additionally, nude mice are not completely immunodeficient, and their immune systems may potentially reject the transplanted cells. Recently, the team led by Professors Anthony Atala and Adam M. Jorgensen reported that after constructing skin-like tissue structures in vivo using multicellular bioprinting, the transplanted epidermal cells survived for 90 days at the wound site but underwent keratinization [41]. In clinical translation, considering that cell survival and functionality can impact the therapeutic potential of C-Ms, if C-Ms prepared from autologous EpiSCs are used for autologous transplantation, the cells would adapt to their own microenvironment, potentially resulting in more significant cell viability and functionality compared with allogeneic transplantation. Wang S, et al. single-cell sequencing data showed that RR formation is closely linked to the proliferative capacity of basal stem cells and the expression of laminin and integrins [29]. Components of dermal-epidermal junction anchoring complexes like integrinα6β4, LM-332, collagen XVII and VII maintain the characteristics of EpiSCs through YAP signaling, thereby controlling their differentiation [23]. In C-Ms-treated wounds, we found a greater number of CK14-positive cells located at RR structures. Transcriptome sequencing analyses suggest that mechanical signals generated by MN may be transduced into chemical signals via laminin, thereby regulating basal stem cell proliferation. We detected a significant activation of LAMA4 in C-Ms-treated wound BM, along with increased expression of ITGB1, suggesting that EpiSCs involved in wound epithelialization responded to MN-induced mechanical stress, and that proliferation of EpiSCs indirectly promoted RR formation. The aim of using bionic MN at the wound site is to provide an RR topology and physical stress that creates a closed space for early EpiSCs adhesion to the BM structures. This was verified by assessing the ki67 activity of EpiSCs in different treatment groups. As epithelialization proceeds, continued proliferation of EpiSCs may generate an inward thrust that further promotes RR formation [20]. However, in the wound environment, continued ECM degradation affects this process. This study preliminarily studied the feasibility of C-Ms to rebuild RRs, but how C-Ms function in the long term and rebuild stem cell niche still needs further investigation.

## Conclusions

The findings of this study are limited by the full-thickness skin defect model in nude mice, such as the inability to fully replicate the human immune system response to cell therapy due to immunodeficiency in nude mice. Secondly, the wound healing mechanisms in nude mice are different from those in humans, which may prevent direct extrapolation of the experimental results to humans. Although we have preliminarily studied the relationship between RRs regeneration and C-Ms remodeling of ECM and stem cell niche, the specific mechanism still needs to be further studied. Clinically, the structural parameters of the RRs of patients are significantly related to age and sites. In follow-up studies, biomimetic RR MNs with universal effects on patients with large areas of skin should be optimized. In addition, pinhole biopsy of the patient’s early skin should be considered in subsequent studies to perform EpiSCs expansion (we have explored the feasibility of this technique in the early stage), with the aim of rapidly realizing the clinical application of biomimetic C-Ms. However, their biosecurity should be a long-term concern. In conclusion, EpiSCs-loaded biomimetic RR MNs (C-Ms) can promote and accelerate the regeneration of the RRs and improve the wound healing quality of full-thickness skin defect wounds in nude mice, and show the possibility of loading autologous EpiSCs-MNs with RRs to treat large-scale burns.

## Materials and methods

### Modeling of RRs structure

Skin samples (0.8 by 0.8 cm) were collected from different body sites (the scalp, the waist, the thigh) after approval from the Ethics Committee of Southwest Hospital of Army Medical University and informed consent from the patients. Samples were obtained from patients of various ages with extensive burns. Subsequently, the collected samples were scanned using the femto-second label-free Imaging technique to observe and model the microscopic structure of the internal cross-sections. The samples were then fixed in 4% paraformaldehyde for 24 h, followed by dehydration and embedding to prepare paraffin samples. Continuous sections were prepared (6 μm thickness, 80 sections in total). The sections were subjected to HE staining and observed under scanning electron microscopy (see the following methods). Statistical analysis of parameters was conducted to simulate a mathematical model of RRs for different ages and body sites.

### Preparation and characterization of microneedles with rete ridge structure

Preparation of GelMA: Gelatin (1 g) was dissolved in 10 mL of deionized water and then stirred continuously at 60 °C for 1 h. Then methacrylic anhydride (0.8 ml) was gradually added to the solution and stirred continuously at 50 °C for 2–3 h. At the end of the reaction the reaction solution was dialyzed using a dialysis membrane (13 kDa) for 4–5 days, replacing the deionized water every 8 h to purify the obtained GelMA product. GelMA (3%, 5% and 7%, w/w) and HAMA (5%, w/w) were dissolved together in PBS (LAP, 0.25%, w/w) to obtain loaded cell microneedle solution. Meanwhile, 10% (w/w) of the GelMA solution was used as the base solution for the microneedles. 1.0 × 10^7 EpiSCs were added to 1 ml cell-loaded microneedle solution. Subsequently, the cell-loaded microneedle solution was transferred into microneedle molds and centrifuged at 1000 ×g for 5 min to achieve complete mold filling. A 100 µL substrate solution was layered onto the mold surface, followed by photoinitiated cross-linking via 365 nm ultraviolet irradiation about 60 s, ultimately generating three-dimensional biomimetic microneedle encapsulating viable cellular components.

### Scanning electron microscopy (SEM) observation

Cryo-lyophilized hydrogel specimens were subjected to gold sputter coating (Au layer thickness: 15 ± 5 nm) to ensure surface conductivity. Subsequent morphological characterization was conducted using field-emission scanning electron microscopy (Gemini 300, Carl Zeiss AG) at 1000× magnification.

### Rheological characterization of biomimetic MNs

The viscoelastic properties of photo-crosslinked biomimetic MNs were systematically evaluated across varying polymer concentrations (3, 5, and 7% w/w GelMA) and curing durations (45, 55, 65 and 75 s). Cylindrical specimens (20 × 5 mm) underwent triple-interval testing using a rotational rheometer: (1) Time-dependent modulus evolution (1% strain, 1 Hz, 25 °C, 0.5 mm gap) over 1,000 s; (2) Frequency-dependent behavior (0.1–100 rad/s, 1% strain); (3) Strain amplitude response (0.1–100%, 10 rad/s); and (4) Shear-thinning characteristics (0.1–100/s).

### Swelling ratio and degradation in vitro

The swelling and enzymatic degradation kinetics of the bionic microneedles were examined separately. The initial mass of the lyophilized microneedles (M0) was first weighed, and then they were immersed in saline at room temperature for 0–48 h and the swelling mass of the microneedles (M1) was recorded. In addition, the mass of the photocrosslinked microneedles was recorded as M2, and then they were immersed in saline solution containing type II collagenase (2.5 U/mL) at 37 °C for 0–48 h, and the microneedles were removed at different time points to record the shape and mass of the microneedles (M3). Finally, the water swelling and enzymatic degradation curves of the microneedles were counted separately.

### In vitro biocompatibility

CCK-8 and Live/Dead Staining: Crosslinked hydrogel specimens (5H, 5H-3G, 5H-5G, 5H-7G) were individually immersed in DMEM (Gibco™, 10% FBS v/v) and equilibrated at 37 °C under 5% CO2 for 24 h to obtain extraction solutions. NIH/3T3 fibroblasts (1.5 × 10^4 cells/mL) were seeded into 96-well plates (100 µL/well) and pre-cultured in standard conditions (37 °C, 5% CO2) for 24 h. Culture medium was aspirated and replaced with hydrogel extracts (100% v/v) after 24 h. CCK-8 and Live Cell Staining Kit (Biotronix) were used separately to treat the cells separately. The absorbance value at 450 nm was determined by enzyme marker. The cell morphology was observed by laser confocal microscopy. All operations were performed with reference to the kit instructions.

### In vivo biocompatibility

Twelve male C57BL/6 mice (5–6 weeks, Army Medical University Animal Center, SYXK(Yu)20170002) were randomized into four groups. Methacrylated hydrogel constructs (5H-5G, 8 mm diameter × 0.2 mm thickness) were subcutaneously implanted following sodium pentobarbital anesthesia (1% w/v, i.p.). Bilateral implantation sites were created via midline dorsal incision (1 cm) under aseptic technique, with wound closure using polypropylene sutures. Specimens including peri-implant skin, residual hydrogels, and major organs (heart, liver, spleen, lung, kidney) were harvested at predetermined intervals (7, 14, 21, 42 days, *n* = 3/timepoint). Tissues underwent 24-hour fixation in 4% paraformaldehyde prior to paraffin embedding and hematoxylin-eosin staining. Hydrogel degradation kinetics were determined through gravimetric analysis of explanted specimens. For hemocompatibility assessment, refer to the previous work for specific experimental steps [32]. In addition, collected skin samples from the transplantation site at 42 days, and extracted the total RNA. Then, qPCR to detect the expression levels of TNFα and IL1β.

### Biological effects of episcs and biomimetic MNs

The C-Ms were subjected to viability analysis following 24-hour culture under standard conditions (37 °C, 5% CO_2_). Spatial cellular distribution within the constructs was visualized via laser scanning confocal microscopy after fluorescent labeling (Live Cell Staining Kit, Beyotime). Hydrogel extracts (5H-5G) were prepared to evaluate cell responses, with EpiSCs (1.5 × 10⁴ cells/mL) seeded in 96-well plates (100 µL/well). Proliferation kinetics were quantified at 24–72 h intervals using CCK-8 assays (Beyotime). Parallel 6-well cultures exposed to extracts for 48 h underwent flow cytometric profiling (FACS Canto II, BD Biosciences) for surface markers: FITC- CD71 (1:200, BioLegend, b253418) and PE-CD49f (1:500, BD 8198762). qPCR analysis compared gene expression profiles between extract-treated and control groups, following RNA extraction and reverse transcription protocols. The specific steps were shown in the supplementary information.

### Regenerative efficacy evaluation of cell-laden microneedle constructs in nude mice wound healing models

Forty-eight male nude mice (7–8 weeks, sourced from the Army Medical University Experimental Animal Center) were randomized into four groups: control, cell suspension (3 × 10⁵ EpiSCs/cm²), MN scaffold, and C-M. Under sodium pentobarbital anesthesia (1% w/v, i.p.), bilateral 8 mm full-thickness excisional wounds were surgically created on dorsal regions. Constructs were topically administered according to group allocation, with wound beds protected using sterile saline-moistened gauze dressings. Specimens were harvested at postoperative days 7, 14, 21 (*n* = 4/group/timepoint), bisected for parallel histomorphometric and transcriptomic analyses. Tissues designated for RNA sequencing underwent immediate flash-freezing prior to shipment to Meiji Biomedical Co., Ltd. (Illumina NovaSeq platform). Corresponding samples were fixed in 4% paraformaldehyde (24 h), paraffin-embedded, and sectioned (6 μm) for histological processing (hematoxylin-eosin, Masson’s trichrome, Picrosirius red staining).

### Longitudinal tracking of episcs

EpiSCs were fluorescently labeled with DiI (Beyotime; 5 µg/mL, 37 °C, 15 min) and engineered into C-Ms. These C-Ms were implanted into full-thickness skin defects of nude mice (7-week-old), with wound samples harvested at post-implantation (7, 14, and 21 days, *n* = 6/group). Tissue specimens were cryosectioned (8 μm), counterstained with DAPI (Sigma), and analyzed via confocal microscopy to track EpiSCs’ engraftment. All procedures complied with institutional animal care protocols.

### Immunofluorescence staining

Paraffin-embedded sections underwent antigen retrieval using tris-EDTA buffer (pH 9.0) at 100 °C for 10 min, followed by cooling to ambient temperature. Non-specific binding sites were blocked with 5% goat serum (Sigma-Aldrich) for 30 min. Primary antibodies were applied overnight at 4 °C: rabbit anti-CK14 (HA720135F, 1:200); rat anti-Laminin-4 (YT2526, 1:1000); mouse anti-Integrinβ1 (YT2367, 1:1000); rabbit anti-CD31 (ab9498, 1:1000); iFluor 647-conjugated anti-Ki67 (HA720163F, 1:200); FITC-anti-fibronectin (ab314679, 1:500); Alexa Fluor 555-anti-TGFβ1 (ab313730, 1:500). After PBST washes (3 × 5 min), species-matched Alexa Fluor secondary antibodies (1:500) were incubated for 2 h at 25 °C. Nuclear counterstaining employed DAPI (1 µg/mL) prior to confocal imaging (LSM 980, Zeiss) under standardized acquisition parameters.

### Real-time fluorescence quantitative PCR and mRNA-seq analysis

After incubating the C-Ms for 24 h, we extracted total RNA for real-time fluorescence quantitative PCR. The detailed methods and primers are provided in the supplementary materials and method. Wound skin samples were collected from the transplanted skin tissues of nude mice (Control, MN, and C-M) on days 7, 14 and 21. Total RNA was then extracted for qPCR and mRNA-seq analysis. Detailed methods are provided in Supplementary materials and methods.

### Statistical analysis

Experimental data were statistically analyzed using GraphPad Prism 9.0. Data from each group were expressed as mean ± standard deviation. If the data satisfied normality and homogeneous variance, then one-way analysis of variance (ANOVA) and post-hoc test were used. Otherwise, Kruskal-wallis test and Dunn’s multiple comparisons were used. *p* < 0.05 was considered statistically significant.

## Electronic supplementary material

Below is the link to the electronic supplementary material.

Supplementary Material 1

## Data Availability

The data are available from the corresponding author upon reasonable request.

## Abbreviations

EpiSCs: Epidermal stem cell
RRs: Rete ridges
C-Ms: Microneedles loaded with EpiSCs
ECM: Extracellular matrix
FAK: Focal adhesion kinase
BM: Basement membrane
MNs: Microneedles
Ctrl: Control
DEGs: Differentially expressed genes
KEGG: Gene function enrichment analysis
GSEA: Gene set enrichment analysis
