# Supplementary material for: Cell-laden biomimetic microneedles reconstruct skin rete ridge and stem cell niche
Source: J Nanobiotechnology. 2025 Jun 4;23:415. doi: 10.1186/s12951-025-03430-x (PMC12135421; doi:10.1186/s12951-025-03430-x)
Supplement: Supplementary file 1 — Supplementary Material 1 [file 12951_2025_3430_MOESM1_ESM.docx]

**Cell-laden biomimetic microneedles reconstruct skin rete ridge and stem cell niche**

Xiaohong Zhao^1†^, Zongze Wu^2†^, Yicheng Guo^1^, Lei Pu^1^, Zixuan Pei^1^, Yuanyuan Liu^1^, Biao Hou^2^, Songlin Xie^2*^, Gaoxing Luo^1*^, Rixing Zhan^1*^

^1^Institute of Burn Research, State Key Laboratory of Trauma and Chemical Poisoning, Southwest Hospital, the Third Military Medical University (Army Medical University), Chongqing 400038, China

^2^Department of Hand and Foot Microsurgery, The affiliated Nanhua Hospital, Hengyang Medical College, University of South China, Hengyang 421002, China.

^†^These authors contributed equally to this work.

^*^Corresponding author.

E-mail addresses: [zhanrixing@tmmu.edu.cn](mailto:zhanrixing@tmmu.edu.cn) (Rixing Zhan), [logxw@tmmu.edu.cn](mailto:logxw@tmmu.edu.cn) (Gaoxing Luo), [xiesonglin0929@163.com](mailto:xiesonglin0929@163.com) (Songlin Xie).

**This file includes:**

Supplementary Materials and Methods

Supplementary Figures S1 to S6

**Supplementary** **Materials and Methods**

**Real-time fluorescence quantitative PCR**

EpiSCs and skin tissue samples were isolated through sequential phosphate-buffered saline (PBS, pH 7.4) rinses. Total RNA was extracted using the RNAprep Pure Cell/Bacteria Kit (TIANGEN Biotech Co., Ltd., Beijing) with subsequent spectrophotometric quantification (NanoDrop 1000, Thermo Fisher Scientific; A260/A280 ratio ≥1.8). Complementary DNA synthesis was performed via reverse transcription using PrimeScript RT Master Mix (Takara Bio, Dalian) followed by 1:10 dilution in nuclease-free water. Quantitative PCR amplifications were conducted in triplicate using TB Green Premix Ex Taq II (Takara Bio) on a CFX96 Real-Time system (Bio-Rad Laboratories) under standardized parameters: initial denaturation (95°C, 30 s); 40 cycles of 95°C (5 s)/60°C (30 s). Target genes included cell cycle regulators (p16INK4a, TP53, CDK4), oncogenic markers (KRAS, PRB, MYC), ECM markers (ITGβ1, Col1a1, LAMA4, FN1), FAK markers (PTK2), Wnt markers (Ctnnb1, Axin2), ERK markers (CCND1), and others (Notch1, TGFβ1, TNFα, IL1β), with NCBI accession numbers provided in Supplementary Table S1), normalized to glyceraldehyde-3-phosphate dehydrogenase (GAPDH). Relative quantification employed the 2^−ΔΔCt^ method with inter-run calibrators and negative controls. Melt curve analyses confirmed amplification specificity, with threshold cycle values recorded when fluorescence exceeded baseline by 10 standard deviations.

Table 1. Primers sequences used in RT- PCR.

| **Gene** | **Primer sequences** |
| --- | --- |
| P16 | F: 5’-cgcgatgtcgcacggta-3’ |
|  | R: 5’-aatcggggatgtctgaggga-3’ |
| P53 | F: 5’-cctctccccagccaaagaag-3’ |
|  | R: 5’-tctcggaacatctcgaagcg-3’ |
| CDK4 | F: 5’-ttgtggccctcaagagtgtg-3’ |
|  | R: 5’-cagtcgcctcagtaaagcca-3’ |
| Kras | F: 5’-tcgacacagcaggtcaagag-3’ |
|  | R: 5’-caaagaaagccctccccagt-3’ |
| PRB | F: 5’-acatctcccaggagagtcca-3’ |
|  | R: 5’-ctgcttttgcattcgtgttcg-3’ |
| MyC | F: 5’-ccctccactcggaaggacta-3’ |
|  | R: 5’-gctggtgcattttcggttgt-3’ |
| ITGβ1 | R: 5’-gatccacaaaccgcaacct-3’ |
|  | F: 5’-tggtcagcaacgcatatctgg-3’ |
| Col1a1 | R: 5’-ccacgtctcaccattgggg-3’ |
|  | F: 5’-gctcctcttaggggccact-3’ |
| PTK2 | R: 5’-ctcgatctctcgatgagtgct-3’ |
|  | F: 5’-gagtacgtccctatggtgaagg-3’ |
| LAMA4 | R: 5’-aggattcgtactgttaccgtca-3’ |
|  | F: 5’-cagcgccaatgctacctgt-3’ |
| FN1 | R: 5’-gcccagtgagtttcagcaaagg-3’ |
|  | F: 5’-atgtggacccctcctgatagt-3’ |
| TGFβ1 | R: 5’-ctggcgagccttagtttggac-3’ |
|  | F: 5’-ccacctgcaagaccatcgac-3’ |
| Notch1 | R: 5’-tcgttgttgttgatgtcacagt-3’ |
|  | F: 5’-gatggcctcaatgggtacaag-3’ |
| Ctnnb1 | R: 5’-catctagcgtctcagggaaca-3’ |
|  | F: 5’-cccagtcttcacgcaagag-3’ |
| Axin2 | R: 5’-gagtgtaaagacttggtccacc-3’ |
|  | F: 5’-aacctatgcccggtttcctcta-3’ |
| CCND1 | R: 5’-acttgaagtaagatacggagggc-3’ |
|  | F: 5’-gcgtaccctgacaccaactc-3’ |
| TNFα | R: 5’-cgatcaccccgaagttcagtag-3’ |
|  | F: 5’-caggcggtgcctatgtctc-3’ |
| IL1β | R: 5’-tggatgctctcatcaggacag-3’ |
|  | F: 5’-gaaatgccaccttttgacagtg-3’ |
| GAPDH | F: 5’-cctggcacccagcacaat-3’ |
|  | R: 5’-gggccggactcgtcatac-3’ |

**mRNA-seq analysis**

Wound skin samples were collected from the transplanted skin tissues of nude mice (Control, MN, and C-M) on days 7 and 14. The skin tissues were rapidly frozen in liquid nitrogen (n=3/group at all time points). RNA Extraction and Library Preparation: Total RNA from the samples was extracted using Trizol reagent according to the manufacturer's protocol. RNA quality and quantity were assessed using the Agilent 2100 Bioanalyzer to obtain the RNA Integrity Number, and strand-specific RNA-seq libraries were constructed. Sequencing: The qualified libraries were sequenced on the Illumina NovaSeq 6000 platform. Clean Data for each sample reached more than 5.97Gb, and the Q30 base percentage was more than 95.69%. Data Preprocessing: Raw sequencing reads were subjected to quality control using FastQC to assess read quality. Quantification of Gene Expression: The raw read counts were normalized using FPKM, or DESeq2’s median of ratios to account for differences in sequencing depth and gene length. Differential Expression Analysis: Differentially expressed genes (DEGs) between treated vs. control were identified using DESeq2. To account for multiple testing, the raw p-values were adjusted using the Benjamini-Hochberg procedure with an FDR cutoff of 5%. Significantly differentially expressed genes were defined as those with an adjusted p-value (q-value) < 0.05 and absolute log_2_ fold change > 1. To explore the biological functions of the DEGs, Gene Ontology (GO) and Kyoto Encyclopedia of Genes and Genomes (KEGG) pathway enrichment analyses were performed, and significantly enriched terms (p-value < 0.05) were visualized. Volcano plots and heatmaps were generated using the Majorbio cloud platform. Gene set enrichment analysis (GSEA) was conducted using GSEA software on the Majorbio cloud platform.”

**Supplementary figure**

**
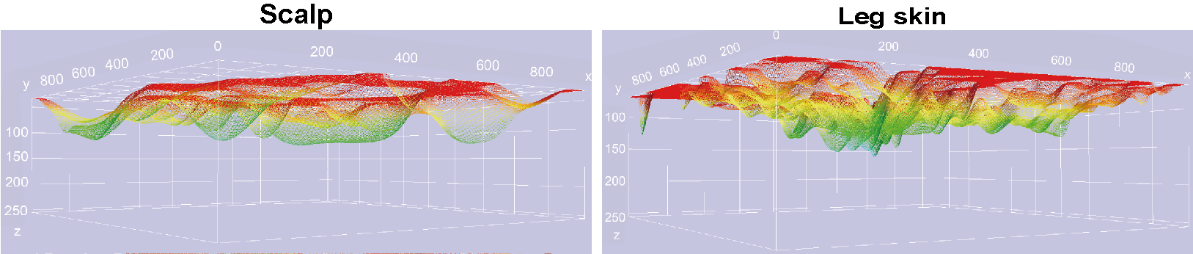
**

**Fig. S1** 3D digital modeling of the rete ridges structure of scalp and leg skin.

**
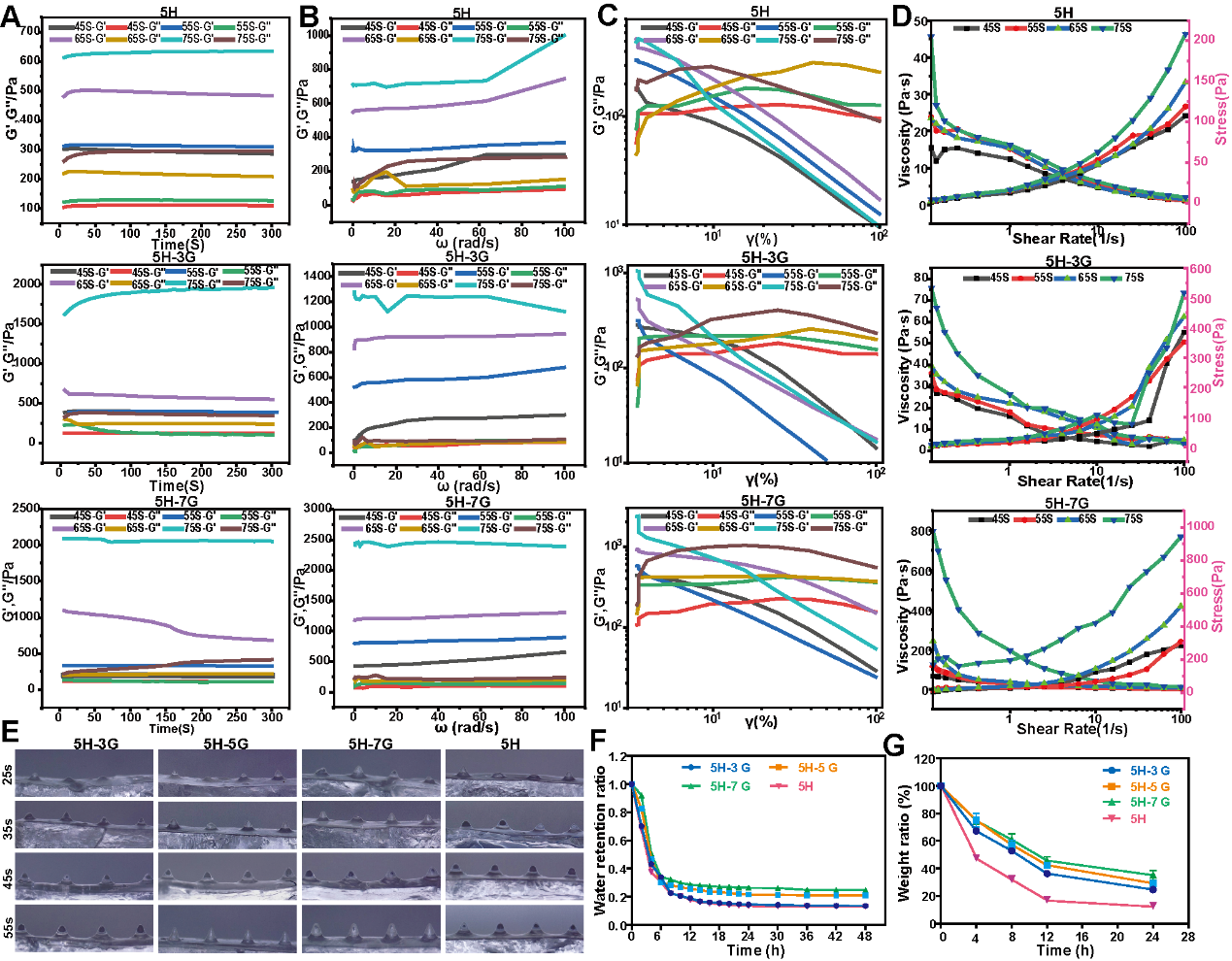
**

**Fig. S2** Evaluated the physical properties of MNs. **(A)** Oscillation time sweep curves related to 5H-3G, 5H-7G and 5H. **(B)** Oscillation frequency sweep curves related to 5H-3G, 5H-7G and 5H. **(C)** Oscillation strain sweep curves related to 5H-3G, 5H-7G and 5H. **(D)** Flow frequency sweep curves related to 5H-3G, 5H-7G and 5H. (**E**) Formation of microneedles under different photo-crosslinking times. (**F**) Degradation of cross-linked 45 s MNs in type II collagenase solution in vitro. (**G**) Water retention properties of MNs at 45 s.

**
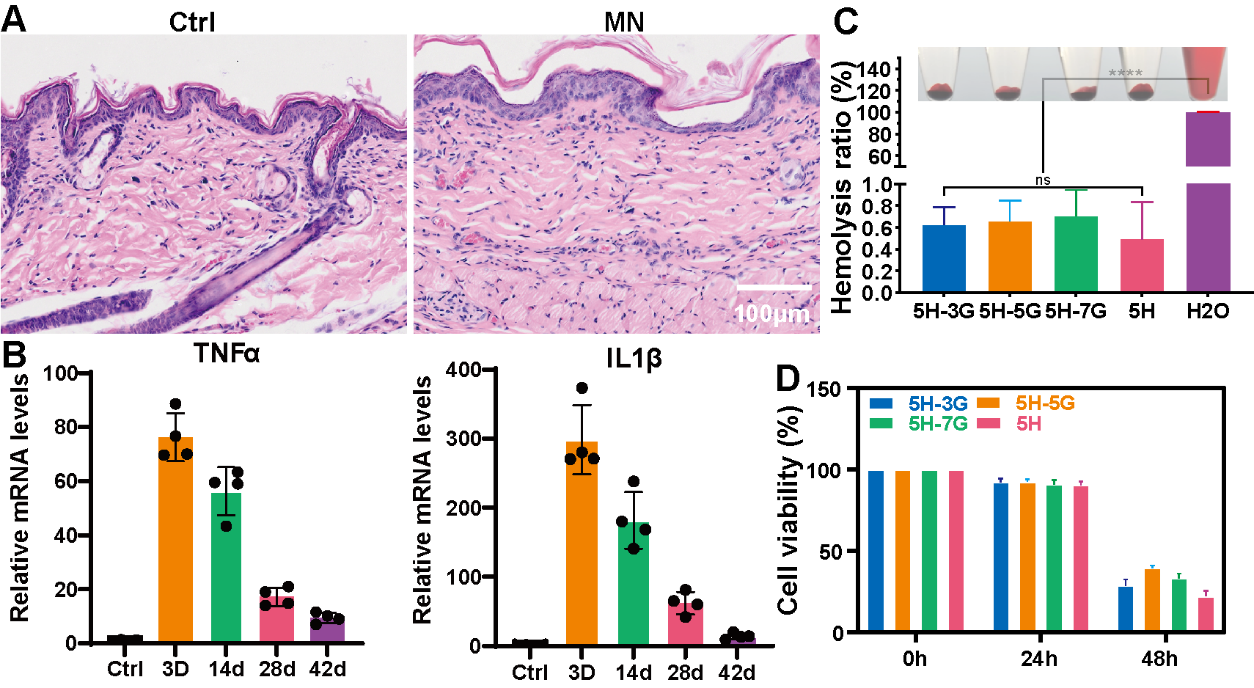
**

**Fig. S3** Evaluated the biosafety of bionic RR MNs. (**A**) HE staining results of the Microneedle subcutaneous graft site skin on day 42. (**B**) We performed real-time quantitative PCR to assess the expression levels of IL1β and TNFα genes in the MN and Ctrl group. (**C**) Hemolysis of microneedle extract. (**D**) The proportion of live epidermal stem cells in the MNs after 48 h was determined.

**
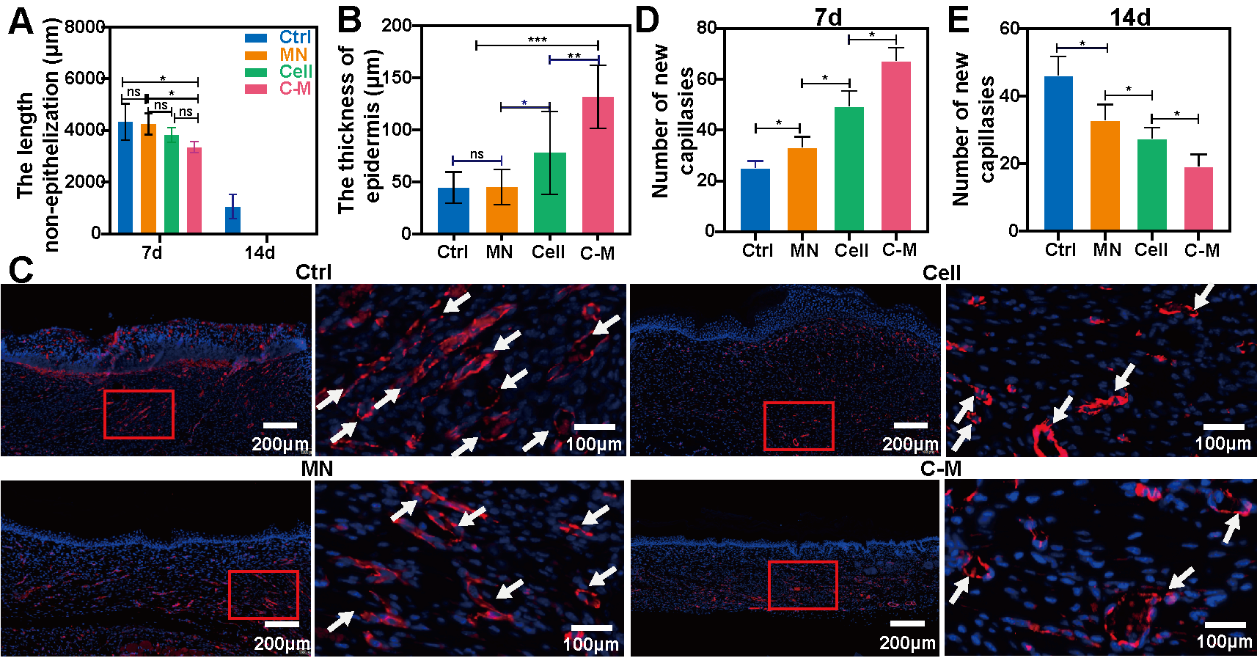
**

**Fig. S4** C-Ms accelerate wound healing, rapid vascularization, and promote skin RRs regeneration. (**A**) Statistical results of epithelialization on days 7 and 14 in each group. (**B**) Statistical results of the thickness of new epidermis on day 21 in each group. (**C**) On day 14, Alexa Fluor® 555 anti-mouse CD31 immunostaining results of wounds in each group. Red boxes and white arrows represented new blood vessels. (**D** and **E**) Statistics on the number of new blood vessels on days 7 and 14, respectively. (**P* < 0.05).

**
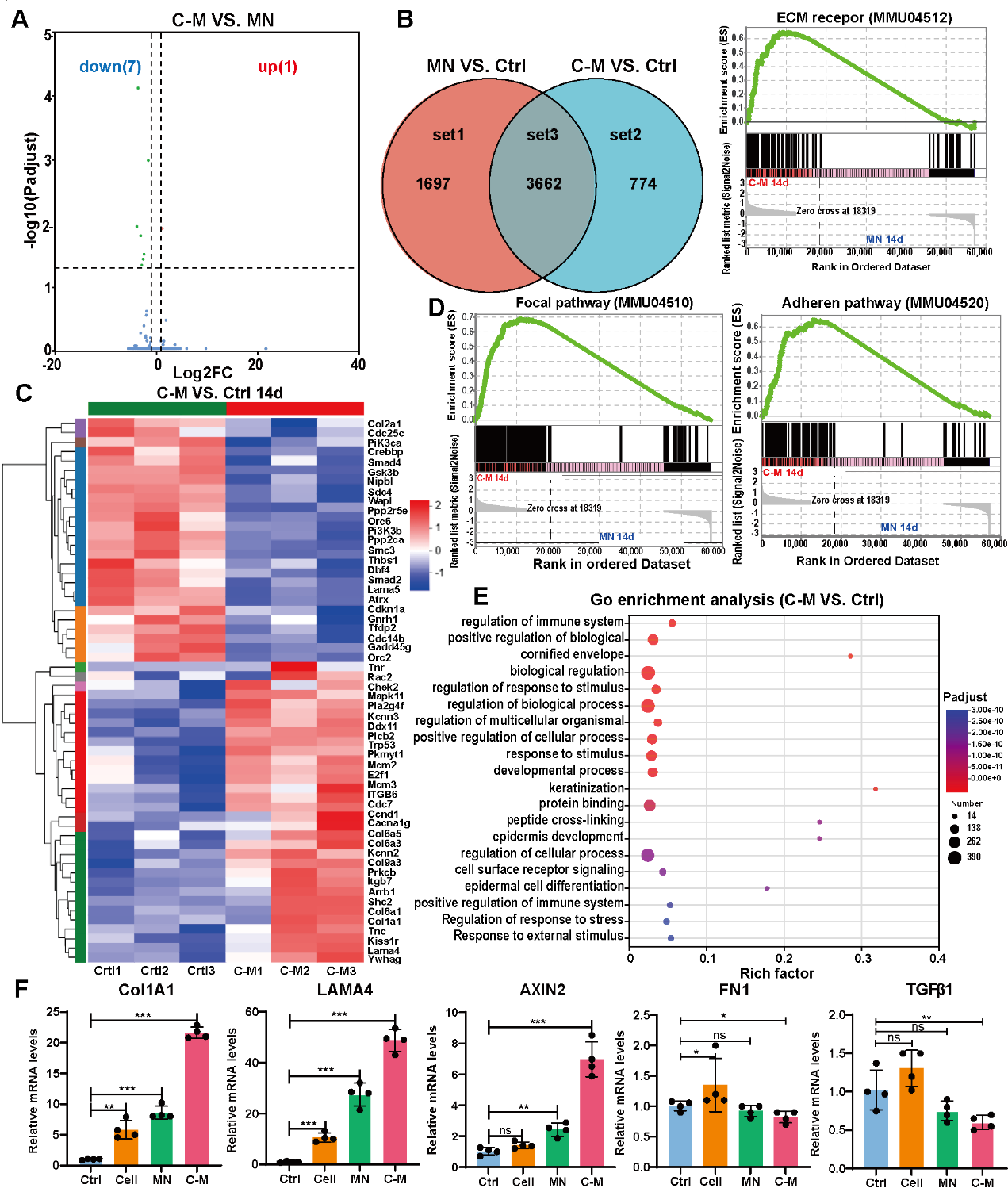
**

**Fig. S5** C-Ms promotes skin RRs formation and provides niche for EpiSCs. (**A**) The volcano map shows up-regulated and down-regulated genes identified 14 days after treatment with MN and treatment with C-M. (FC ≥ 2.0, *P* < 0.05). n = 3. (**B**) Using a Venn diagram to display the differential genes in the control, MN, and C-M groups. (**C**) The heat map displays the results of differential genes between the control group and the C-M group. (**D**) GSEA analysis of RNA-seq data for C-M vs. MN. (**E**) GO enrichment analysis of genes significantly changed for C-M vs. Control. (FC ≥ 2.0, *P*< 0.05). (**F**) The expression of representative genes in skin tissues was detected by qPCR. (**P* < 0.05).

**
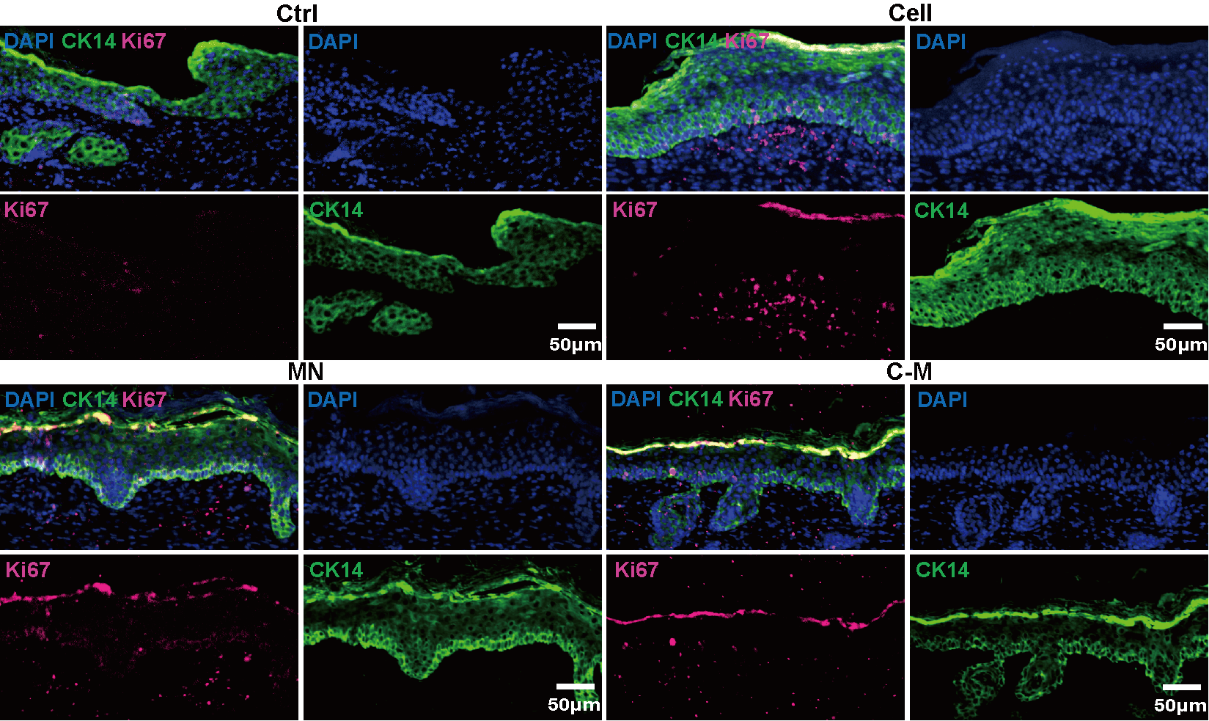
**

**Fig. S6** The representative immunofluorescent staining results were shown of control, cell only, MN only and C-M only 14 days after transplantation. Top row: Anti-mouse laminin4 (red), DAPI (blue) and anti-CK14 (green). Middle row: Anti-mouse ITGB1 (pink), DAPI (blue), and anti-CK14 (green). Bottom row: Anti-mouse Ki67 (pink), DAPI (blue), and anti-CK14 (green).
